# Reply to “A better interpretation of data regarding the opioid switching to methadone”

**DOI:** 10.1186/s12904-023-01162-z

**Published:** 2023-06-07

**Authors:** Haiying Ding, Yu Song, Wenxiu Xin, Jiao Sun, Like Zhong, Qinfei Zhou, Chaoneng He, Liyan Gong, Luo Fang

**Affiliations:** 1 Department of Pharmacy, Zhejiang Cancer Hospital, Institute of Basic Medicine and Cancer (IBMC), Chinese Academy of Sciences, Hangzhou, China; 2 Department of Rare Cancer & Head and Neck Medical Oncology, Zhejiang Cancer Hospital, Institute of Basic Medicine and Cancer (IBMC), Chinese Academy of Sciences, Hangzhou, China

**Keywords:** Cancer pain, Opioid switching or rotation, Methadone, Palliative care

## Abstract

In our article ?Methadone switching for refractory cancer pain’ (BMC palliative care, 2022) we explore the efficacy, safety and economics of methadone in treatment of patients with refractory cancer pain in China. Professor Mercadante provided a better interpretation of data regarding the opioid switching to methadone in the Matters Arising. In this article, we answered the questions in Mercadante et al.’s comments one by one.

We have read with interest the “Matters Arising” text of Professor Mercadante, in which he comments on some parts of our recent paper entitle “Methadone switching for refractory cancer pain” [1]. As he interpreted, several studies in the last 30 years have demonstrated the efficacy of methadone for refractory cancer pain [2–4]. However, no data related to methadone therapy for cancer pain in the Chinese population has been published to date. It was generally accepted that extensive interindividual variability exists in both pharmacokinetics (PK) and pharmacodynamics (PD) of methadone, which can be partially explained by the genetic variants in the CYP enzymes (CYP3A, CYP2D6, CYP2C19 and CYP2B6). It is known that east Asians show a different activity of these CYP enzymes in comparison with Caucasians owing to the demographic, physiological and genetic differences [5, 6]. Guo et al. [7] indicated that the genetic variants along with other variables determined the disparity in PK and PD profile of methadone between Chinese and Western population. Therefore, it is urgent to explore the use of methadone in Chinese cancer pain patients considering the difference in dosing regimens, efficacy and safety profiles of methadone from other population. Our study aims to explore the efficacy, safety and economics of methadone in treatment of patients with refractory cancer pain in China.

First, Professor Mercadante wondered if patients were uncontrolled pain given the fact that the median pain score was 4/10. It is well known that the frequency of breakthrough pain (BTP) requiring supplemental doses is as important as pain score in pain evaluation [8]. “poor pain control”or “uncontrolled pain” can be defined as pain that does not met the patient goals, including uncontrolled persistent pain and/or uncontrolled BTP [8, 9]. In our study, 30% of the included patients experienced unsatisfactory pain relief who continued to suffer uncontrolled breakthrough pain (frequency of BTP ≥ 3 in 24 hours) with NRS < 4. According to expert consensus from the Chinese Committee of Rehabilitation and Palliative Care (CRPC), background long-acting opioid dose can be increased if BTP is uncontrolled. For patients with refractory pain which showed poor response to repeated opioid dose escalation, switching to methadone might be a good strategy. Our study showed that median pain score significantly decreased from 4.0 to 2.0 (p < 0.001) and median daily frequency of BTP from 3.0 to 0.0 (p < 0.001) after switching to methadone [1]. For patients with uncontrolled BTP, decrease in frequency of BTP is more clinically meaningful than the decrease in pain intensity. In addition, satisfaction with pain control was clinically improved for patients treated with methadone as observed by our pain specialists, which was consistent with the statistically significant differences in our study.

Second, as Professor Mercadante interprets, it is difficult to understand why some patients were assigned to 3-day switch (3DS) or stop and go (SAG) attributed to the design of retrospective study. And the modality of switching to methadone in our study reflects the practice of the local institution. We accept the limitation in incomprehensibile choice of SAG or 3DS in our study. However, it is imperative to conduct practice or exploration and report the results on methadone treatment in Chinese patients with cancer pain. Subjected to law and drug production restrictions, the exploration of methadone in treatment of patients with refractory cancer pain have just begun in China in recent years.

We started to use methadone to treat refractory cancer pain in 2016. In the course of exploration, we have tried both SAG and 3DS strategy. The SAG strategy has gained popularity for the obvious pharmacokinetic advantages [10]. Our clinical experience also suggests that SAG is efficient for patients with conventional dose of opioids. However, for patients with high dose of opioids, especially those with doses above 300 mg of oral morphine equivalents (OME)/day, 3DS may have an advantage with less adverse reactions (e.g., headache, sweating) and better medication adherence. There is no doubt that this study, as a single-center retrospective study, is limited in strength, and cannot conclude that 3DS is superior. Whereas, our practice results provide confidence in the use of methadone in the Chinese population. More and more clinicians in China are trying to switch to methadone for the treatment of refractory cancer pain, which greatly encourages our team to further study this field.

Finally, Mercadante S. suggested that the design and results of the randomized controlled study quoted in our study have some limitations [11, 12]. The low number of patients, the logistics of the study and the lack of flexibility in methadone doses, particularly in the SAG group, does not allow to draw the conclusion that 3DS works better than SAG. We agree with these points. A more flexible use of both SAG and 3DS strategy and strict clinical observation to change doses according to the clinical response may provide the optimal treatment.

There is currently no consensus on recommendation for the most effective and safest method for switching to methadone from other opioids. McLean S. et al. reviewed the available evidence regarding methods of rotation to methadone [13]. Twenty-five studies were identified: One randomized clinical trial comparing the 3DS and rapid conversion (RC) SAG methods [11], two other studies examining the 3DS method, 10 studies examining the RC SAG method, 9 studies examining the ad libitum SAG method, and 3 studies describing other methods. Taking limitations in the evidence into account, there was a trend toward excess AEs using the SAG method, in comparison to the 3DS methods. Nonetheless, most evidence was of low quality.

We agree that there is no reason to maintain a drug in patients with high doses of opioid experiencing poor pain control. However, the treatment level varies greatly between countries or regions. Many cancer pain patients were prescribed high dose opioids (above 300 mg OME/day) and the doses might even be increased to > 1000 mg OME/day in China. Switching to methadone may be an effective strategy for these patients with uncontrolled pain. 3DS is based on the concerns about safety of switching to methadone. It showed better tolerance especially in patients prescribed with high doses of other opioids.

In our center, SAG was also adopted for patients with high dose of OME. However, according to the NCCN guideline for cancer pain [8], the recommended initial dose of methadone was quite low for these patients considering the safety, which may lead to insufficient analgesic at the first few days and requirement of frequent supplemental doses of short-acting analgesics. Methadone dose should be gradually increased to achieve satisfactory pain control, thereby prolongating the length of hospital stay.

According to the published literature and our local practice, we still can not conclude which switching method for methadone is more superior. Therefore, a rigorously designed randomized controlled study may be helpful in determining the optimal rotation method for specific patients and guiding future clinical practice.

## Data Availability

NA.

## Abbreviations

PK: Pharmacokinetics
PD: Pharmacodynamics
BTP: Breakthrough pain
3DS: 3-day switch
SAG: Stop and go
OME: Oral morphine equivalents
RC: Rapid conversion
